# Surveillance for parasites in unaccompanied minor refugees migrating to Germany in 2015

**DOI:** 10.3205/dgkh000265

**Published:** 2016-03-01

**Authors:** Ursel Heudorf, Maria Karathana, Bernhard Krackhardt, Meike Huber, Peter Raupp, Christian Zinn

**Affiliations:** 1 Public Health Department, Infectiology and Hygiene, Frankfurt/Main, Germany; 2 Public Health Department, Pediatrics, Frankfurt/Main, Germany; 3 Center for Hygiene and Infection Prevention, Ingelheim, Germany

**Keywords:** refugees, unaccompanied minor refugees, parasites

## Abstract

In 2015, most of the refugees arriving in Germany originated from countries with poor hygienic and sanitary conditions. Stool samples of 1,230 minor refugees unaccompanied by adults were investigated for possible parasites. *Giardia lamblia* was by far the most frequently detected parasite (n=165); all other parasites were considerably less frequent and encountered in the following order: *Hymenolepis nana* (n=23), *Entamoeba histolytica* (n=17), *Trichuris trichiura* (n=8), and *Blastocystis hominis* (n=1). *Ascaris lumbricoides* was not detected among any of the screened refugees. Considerable differences in prevalence rates in refugees originating from different countries could be observed.

## Zusammenfassung

Die meisten Flüchtlinge, die im Jahr 2015 in Deutschland eintrafen, kamen aus Ländern mit ungünstigen Hygienebedingungen. In den auf Parasiten getesteten Stuhlproben von 1.230 unbegleiteten minderjährigen Asylsuchenden wurde *Gardia lamblia* am häufigsten (n=165) gefunden. Alle anderen untersuchten Parasiten traten sehr viel seltener mit folgenden Häufigkeiten auf: *Hymenolepis nana* (n=23), *Entamoeba histolytica* (n=17), *Trichuris trichiura* (n=8) und *Blastocystis hominis* (n=1). *Ascaris lumbricoides* konnte in keiner Probe gefunden werden. Zwischen den Asylsuchenden aus verschiedenen Herkunftsländern konnten erhebliche Unterschiede in den Prävalenzraten festgestellt werden.

## Introduction

The current refugee crisis demands European union-wide surveillance [1]. The Robert Koch-Institute in Germany has informed all physicians about possible epidemiologically relevant infectious diseases in conjunction with refugees, including vaccine-preventable diseases such as hepatitis A, influenza, pertussis, measles, mumps and varicella, respiratory and gastrointestinal diseases such as tuberculosis and norovirus gastroenteritis. However, only a single parasitic disease, scabies, was included in this information [2]. 

Most of the refugees in Germany are coming from countries with poor sanitation practices or poor hygienic conditions, especially after the break of the political systems due to terrorism and/or civil war in their country of origin. Therefore it might be assumed that these refugees might harbor parasites. This may not only be a problem, which may have implications in refugee camps with poor sanitation, but may affect health conditions of affected individuals as well. 

## Method

All unaccompanied minor refugees age 16.0 ± 1.2 years and below 18 years arriving in the South of Hesse, a federal state in the south-west of Germany, from January 1^st^ to November 10^th^ 2015, had their stool samples tested for *Giardia lamblia, Entamoeba histolytica, Ascaris lumbricoides, Trichuris trichiura, Hymenolepis nana, *and* Blastocystis*. The analyses were performed in the Institute for Medical Microbiology, Bioscientia, Ingelheim, Germany, according to standard methods (microscopy and ELISA-test; immunoassay).

## Results

Of the 1,230 refugee minors tested, 750 (61%) were coming from Afghanistan, 135 (11%) from Eritrea, 118 (10%) from Somalia, 75 (6%) from Syria, 39 (3%) from Ethiopia, 27 (2%) from Maghreb/North Africa (Morocco, Libya, Algeria), 35 (3%) from Sub-Saharan Africa, 21 (2%) from Middle East (among them 9 coming from Iran, and 9 from Iraq), 11 (1%) from Asia (9 Pakistan, 1 India, 1 Sri Lanka) and 19 from other countries (11 Albania, 3 Kosovo, 3 Ukraine, 1 Armenia, and 1 unknown) (Table 1 (Tab. 1)).

*Giardia lamblia* was by far the most frequently detected parasite (165; 13.4%). All other parasites were less frequent: *Hymenolepis nana* (23; 1.9%), *Entamoeba histolytica/dispar* (17; 1.4%), *Trichuris trichiura* (8; 0.7%), *Blastocystis hominis* (1; 0.1%). Table 1 (Tab. 1) depicts the distribution according to origin of the children/adolescents. Prevalence of *Giardia *sp. cysts was highest in children from Asia (18.2%) and Eritrea (17.8%), whereas *Entamoeba histolytica* was most often detected in refugees from the Middle East (9.5%), and *Trichuris trichiura* was found in 9.1% of those coming from Asia. *Ascaris lumbricoides* was not detected in any of the specimens.

## Discussion

In general, Giardiasis and Amebiasis are frequent in tropical areas with poor sanitation. These diseases are transmitted through contaminated water or food. Amoeba cause acute and chronic intermittent diarrheal diseases. Giardiasis can cause bloody diarrhea, stomach cramping and fever. In severe cases complications such as gastrointestinal perforation and hematogenous abscesses may occur. 

Ascaris infection is one of the most common intestinal worm infections. The geographic distributions of *Ascaris *spp. are worldwide in areas with warm moist climates and are widely overlapping. Therefore it is surprising, that *Ascaris lumbricoides* could not be detected in any of the investigated samples. 

The third most common roundworm of humans is common in warmer areas especially in Asia and to a lesser degree in Africa and South America. *Hymenolepis nana* (dwarf tapeworm) is a cosmopolitan species though occurring most commonly in temperate zones; it is one of the most common cestodes world-wide [3].

None of the refugees tested reported on gastrointestinal symptoms. Most parasites were detected in less than 2% of the refugees. However, more than 10% of the unaccompanied minor refugees exhibited *Giardia lamblia* in their stool specimen. In agreement with this data, first findings of wastewater monitoring showed increased contamination of *Giardia lamblia* in wastewater of a school used as emergency accommodation for about 200 refugees in Frankfurt am Main: 4,600,000 spores/100 l compared to 90,000 spores/100 l in samples of the Frankfurt municipal sewage treatment plant [4]. 

Because parasitic diseases with the exception of Giardiasis are not notifiable in Germany, no comparison to nationwide data can be made. Currently, there is no evidence of an increase of the Giardiasis notifications neither in the federal state of Hesse nor nationwide. Occasionally, employees of the public health office are contacted and asked for advice when individual cases of helminth diseases occur. Reliable data are not available in Germany as there is no mandatory reporting for these conditions. The data may be compared, however, to a small survey of 102 unaccompanied asylum seekers, arriving in Bielefeld, Germany, with about 20% of them being infected with parasites (7.2% Lambliasis, 6.3% Amoebiasis, 7.6% helminthic diseases) [5] and to the survey of 1,203 refugees in California, 2008–2010, with 12.3% of them being infected with parasites [6]. 

Catchpole and Coulombier [1] proposed surveillance for communicable diseases in refugees due to missing vaccination such as influenza, measles and varicella, as well as infections and outbreaks of scabies, diarrhea and meningococcal disease. This might be achieved by assessing the data of notifiable diseases, if the criteria “refugee” is made reportable as well or by special surveys in defined migration centers (i.e. [7], [8]). Riccardo et al. highlighted the current available data in the EU and their limitations [9]. Additional studies with data of screening asymptomatic persons for multidrug resistant organisms [10], [11], or parasites, as shown by the data presented here, seem to be necessary to assess the burden of these organisms and parasites in refugees. Moreover, wastewater surveillance, which is already established in environmental monitoring of polio virus in various countries (i.e. Italy and Switzerland [12], [13]), might be helpful in surveillance of parasites as well. 

## Notes

### Competing interests

The authors declare that they have no competing interests.

## Figures and Tables

**Table 1:**
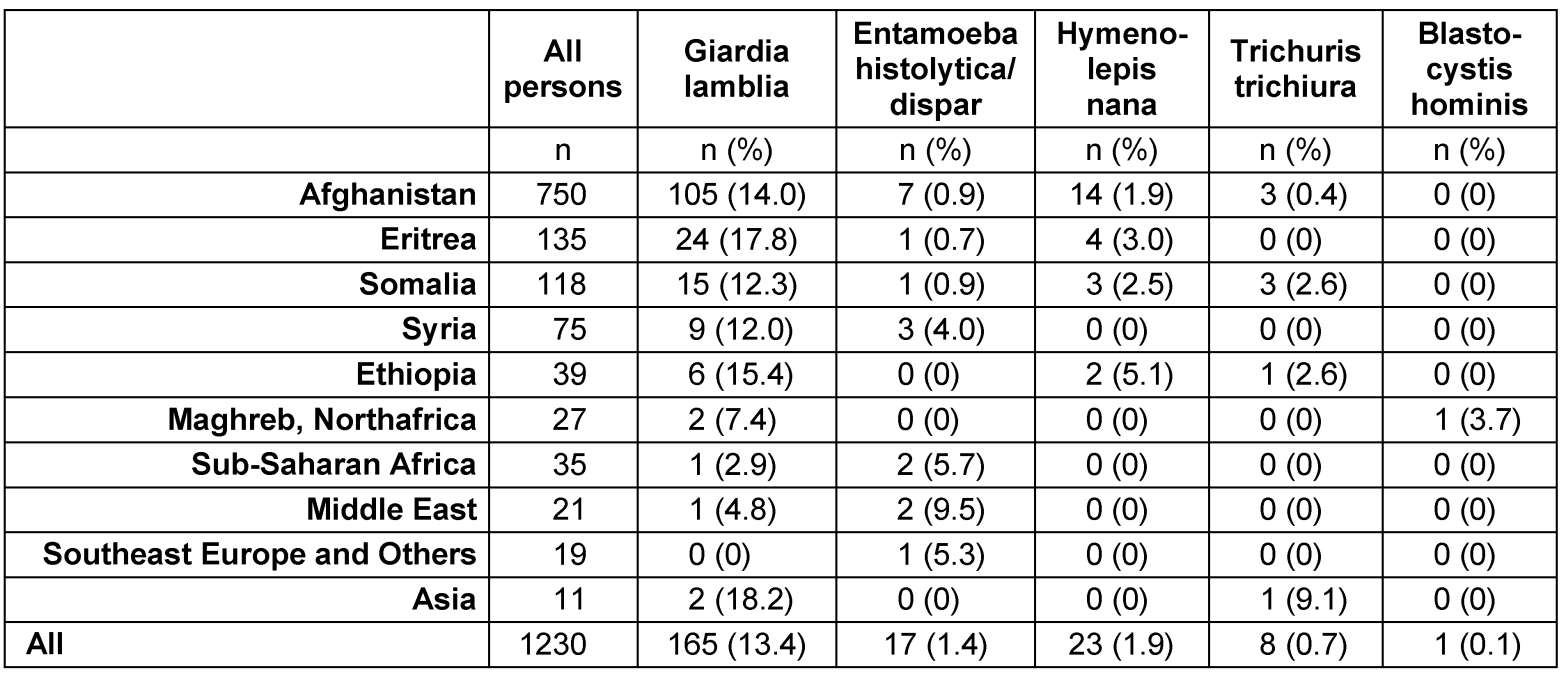
Prevalence of parasites and worms in 1,230 refugee minors from various countries, arrived in Frankfurt, Germany in 2015

## References

[1] Catchpole M, Coulombier D (2015). Refugee crisis demands European Union-wide surveillance!. Euro Surveill.

[2] Beermann S, Rexroth U, Kirchner M, Kühne A, Vygen S, Gilsdorf A (2015). Asylsuchende und Gesundheit in Deutschland: Überblick über epidemiologisch relevante Infektionskrankheiten. Dt Ärztebl.

[3] Mehlhorn H (2008). Encyclopedia of Parasitology.

[4] Exner M Pers. communication; findings No. 33348 from 04th December 2015.

[5] Marquardt L, Krämer A, Fischer F, Prüfer-Krämer L (2016). Health status and disease burden of unaccompanied asylum-seeking adolescents in Bielefeld, Germany: cross-sectional pilot study. Trop Med Int Health.

[6] Chang AH, Perry S, Du JN, Agunbiade A, Polesky A, Parsonnet J (2013). Decreasing intestinal parasites in recent Northern California refugees. Am J Trop Med Hyg.

[7] Napoli C, Riccardo F, Declich S, Dente MG, Pompa MG, Rizzo C, Rota MC, Bella A, The National Working Group 3 (2014). An early warning system based on syndromic surveillance to detect potential health emergencies among migrants: results of a two-year experience in Italy. Int J Environ Res Public Health.

[8] Riccardo F, Napoli C, Bella A, Rizzo C, Rota MC, Dente MG, De Santis S, Declich S (2011). Syndromic surveillance of epidemic-prone diseases in response to an influx of migrants from North Africa to Italy, May to October 2011. Euro Surveill.

[9] Riccardo F, Giorgi Rossi P, Chiarenza A, Noori T, Declich S (2015). Letter to the editor: Responding to a call for action - where are we now?. Euro Surveill.

[10] Reinheimer C, Kempf VAJ, Göttig S, Hogardt M, Wichelhaus TA, O'Rourke F, Brandt C (2016). Multidrug-resistant organisms detected from refugee patients admitted to a German University Hospital. Euro Surveill.

[11] Heudorf U, Krackhardt B, Karathana M, Kleinkauf N, Zinn C (2016). Multidrug-resistant bacteria in unaccompanied refugee minors arriving in Frankfurt am Main, Germany, October to November 2015. Euro Surveill.

[12] Battistone A, Buttinelli G, Fiore S, Amato C, Bonomo P, Patti AM, Vulcano A, Barbi M, Binda S, Pellegrinelli L, Tanzi ML, Affanni P, Castiglia P, Germinario C, Mercurio P, Cicala A, Triassi M, Pennino F, Fiore L (2014). Sporadic isolation of sabin-like polioviruses and high-level detection of non-polio enteroviruses during sewage surveillance in seven Italian cities, after several years of inactivated poliovirus vaccination. Appl Environ Microbiol.

[13] Zurbriggen S, Tobler K, Abril C, Diedrich S, Ackermann M, Pallansch MA, Metzler A (2008). Isolation of sabin-like polioviruses from wastewater in a country using inactivated polio vaccine. Appl Environ Microbiol.

